# Use of pain-related gene features to predict depression by support vector machine model in patients with fibromyalgia

**DOI:** 10.3389/fgene.2023.1026672

**Published:** 2023-03-29

**Authors:** Fengfeng Wang, Chi Wai Cheung, Stanley Sau Ching Wong

**Affiliations:** Department of Anaesthesiology, School of Clinical Medicine, Li Ka Shing Faculty of Medicine, The University of Hong Kong, Hong Kong, China

**Keywords:** chronic pain, gene co-expression, depression, fibromyalgia, principal component analysis, support vector machine

## Abstract

The prevalence rate of depression is higher in patients with fibromyalgia syndrome, but this is often unrecognized in patients with chronic pain. Given that depression is a common major barrier in the management of patients with fibromyalgia syndrome, an objective tool that reliably predicts depression in patients with fibromyalgia syndrome could significantly enhance the diagnostic accuracy. Since pain and depression can cause each other and worsen each other, we wonder if pain-related genes can be used to differentiate between those with major depression from those without. This study developed a support vector machine model combined with principal component analysis to differentiate major depression in fibromyalgia syndrome patients using a microarray dataset, including 25 fibromyalgia syndrome patients with major depression, and 36 patients without major depression. Gene co-expression analysis was used to select gene features to construct support vector machine model. The principal component analysis can help reduce the number of data dimensions without much loss of information, and identify patterns in data easily. The 61 samples available in the database were not enough for learning based methods and cannot represent every possible variation of each patient. To address this issue, we adopted Gaussian noise to generate a large amount of simulated data for training and testing of the model. The ability of support vector machine model to differentiate major depression using microarray data was measured as accuracy. Different structural co-expression patterns were identified for 114 genes involved in pain signaling pathway by two-sample KS test (*p* < 0.001 for the maximum deviation D = 0.11 > D_*critical*_ = 0.05), indicating the aberrant co-expression patterns in fibromyalgia syndrome patients. Twenty hub gene features were further selected based on co-expression analysis to construct the model. The principal component analysis reduced the dimension of the training samples from 20 to 16, since 16 components were needed to retain more than 90% of the original variance. The support vector machine model was able to differentiate between those with major depression from those without in fibromyalgia syndrome patients with an average accuracy of 93.22% based on the expression levels of the selected hub gene features. These findings would contribute key information that can be used to develop a clinical decision-making tool for the data-driven, personalized optimization of diagnosing depression in patients with fibromyalgia syndrome.

## 1 Introduction

Chronic pain, defined as pain lasting at least 3 months, is a major clinical and socioeconomic burden to both patients and society (Queiroz, 2013; Eucker et al., 2022). Fibromyalgia syndrome (FMS) is a chronic pain condition associated with diffuse musculoskeletal pain, cognitive dysfunction, sleep disturbance, somatic symptoms, and psychological distress (Siracusa et al., 2021; Farag et al., 2022; Kumbhare et al., 2022). FMS affects around 5% of global population, and is more prevalent in females (Wolfe et al., 1995; Siracusa et al., 2021). Patients with FMS often suffer from functional disabilities, including impairment with work and activities of daily living (Arnold et al., 2008; Clauw et al., 2011). FMS is associated with psychiatric and psychological disorders, such as depression and anxiety (Bennett et al., 2007; Clauw et al., 2011). Depression is more likely to be present in patients with FMS (Santos et al., 2011), with an incidence ranging from 9.2% to 90% depending on different screening or diagnostic methods (Wilke et al., 2010; Gota et al., 2017). It has been reported that more than half of all FMS patients experienced major depressive disorder during their life-time (Løge-Hagen et al., 2019). Patients with chronic pain who have co-existing depression suffer from greater pain intensity and longer duration of pain, and are less likely to respond to treatment (Bair et al., 2003). Depression is commonly unrecognized in patients with chronic pain, and therefore untreated (Lee et al., 2018). Negating depression in chronic pain patients may increase the chance of treatment failure. Formal diagnosis usually depends on clinical experience and subjective evaluation by psychiatrists. However, physicians managing chronic pain in FMS patients are non-psychiatrists and often lack the expertise to diagnose major depression with a high level of certainty. This leads to low consistency and accuracy, resulting in major depression in FMS patients being missed (Yan et al., 2022). A personalized tool for differentiating FMS patients with major depression by identifying biological predictors in patients with FMS will enhance diagnostic accuracy and improve clinical outcomes.

Support vector machine (SVM) is one promising technique for identifying biological markers, which has been widely used to diagnose and classify various diseases, especially depression. Researchers have developed an integrated analytical algorithm consisting of nuclear magnetic resonance-based metabolomics and least squares-SVM to diagnose depression (Zheng et al., 2017). SVM was applied to predict the efficacy of escitalopram from electroencephalography recordings for treatment of depression (Zhdanov et al., 2020). Furthermore, SVM was used to separate depressed from healthy individuals based on multiple brain network properties, such as diffusion-weighted neuroimaging and graph theory (Sacchet et al., 2015). These studies provide strong evidence that SVM can be applied to predict depression. However, they cannot translate these findings into clinical tools, due to low accuracy of prediction or small sample sizes. To overcome these limitations, the present study attempted to use Gaussian noise to generate a large amount of simulated data for model construction with a high accuracy.

Since pain and depression can cause each other and worsen each other, we hypothesize that pain-related genes can be used to differentiate between those with major depression from those without. In this study, whole genome data analysis was applied to select gene features to construct SVM model combined with principal component analysis (PCA) technique for prediction of depression in patients with FMS. Usually, multiple variables are included in SVM models, leading to a high-dimension, and it is difficult to find the patterns in data with high-dimension. PCA can help reduce the number of data dimensions without much loss of information, and identify patterns in data easily (Jolliffe, 1986). Our proposed method could provide useful information for personalized optimization of diagnosing depression in patients with FMS.

## 2 Materials and methods

### 2.1 Microarray expression data and study subjects

The microarray dataset GSE67311, from publicly available Gene Expression Omnibus (GEO) repository database, was used for analysis in this study (Jones et al., 2016). The data were normalized by Robust Multi-array Average method across the samples. Blood samples were collected from 70 FMS patients and 70 healthy matched controls. The subjects were limited to Caucasian females aged 18 and over. Fibromyalgia patients were diagnosed by a physician with FMS for at least 6 months or longer (Jones et al., 2016). There were 25 FMS patients with major depression, and 36 patients without major depression, which were used to construct the model.

### 2.2 Co-expression analysis and feature selection

Usually, pain and depression can cause each other and worsen each other. The pain signaling system consists of transduction, conduction, synaptic transmission, and modulation, and 114 pain-related genes identified from the microarray dataset were summarized for analysis (Supplementary Table S1) (Julius and Basbaum, 2001; Foulkes and Wood, 2008). We further extracted the expression profiles of these 114 genes from dataset GSE67311 for co-expression analysis according to the methods developed in our previous studies (Wang et al., 2014; Chan et al., 2015; Wang et al., 2015; Wang et al., 2016).

An FMS-specific cutoff point was identified to classify the co-expressed gene pairs into strong and weak co-expression classes. The normal-specific strongly co-expressed pairs were the gene pairs strongly co-expressed only in the healthy individuals, which were disrupted in the FMS group, called disrupted links. The FMS-specific strongly co-expressed pairs were the gene pairs strongly co-expressed only in the FMS group, which were invoked in the FMS group, called invoked links. There were also common weakly co-expressed pairs and common strongly co-expressed pairs. The disrupted links were regarded as the inter-gene linkages maintaining physiological balance in healthy individuals. The invoked links represented the characteristics of the disease and may be the pathogenic alternatives.

The sum of absolute Pearson correlation coefficient (|r|) values for each gene in the co-expression network was calculated, defined as Rsum. Genes with the largest Rsum were called hub genes. Throughout the study, Rsum values were used to select hub genes for depression prediction. Cytoscape is an open source software used to visualize complex (Shannon et al., 2003). ClueGO is a Cytoscape plug-in to annotate large sets of genes by integrating Gene Ontology, KEGG/BioCarta pathways and other databases (Bindea et al., 2009). We applied ClueGO in Cytoscape to interpret the biological function of selected hub gene features.

### 2.3 Support vector machine model construction

**FIGURE 1:**
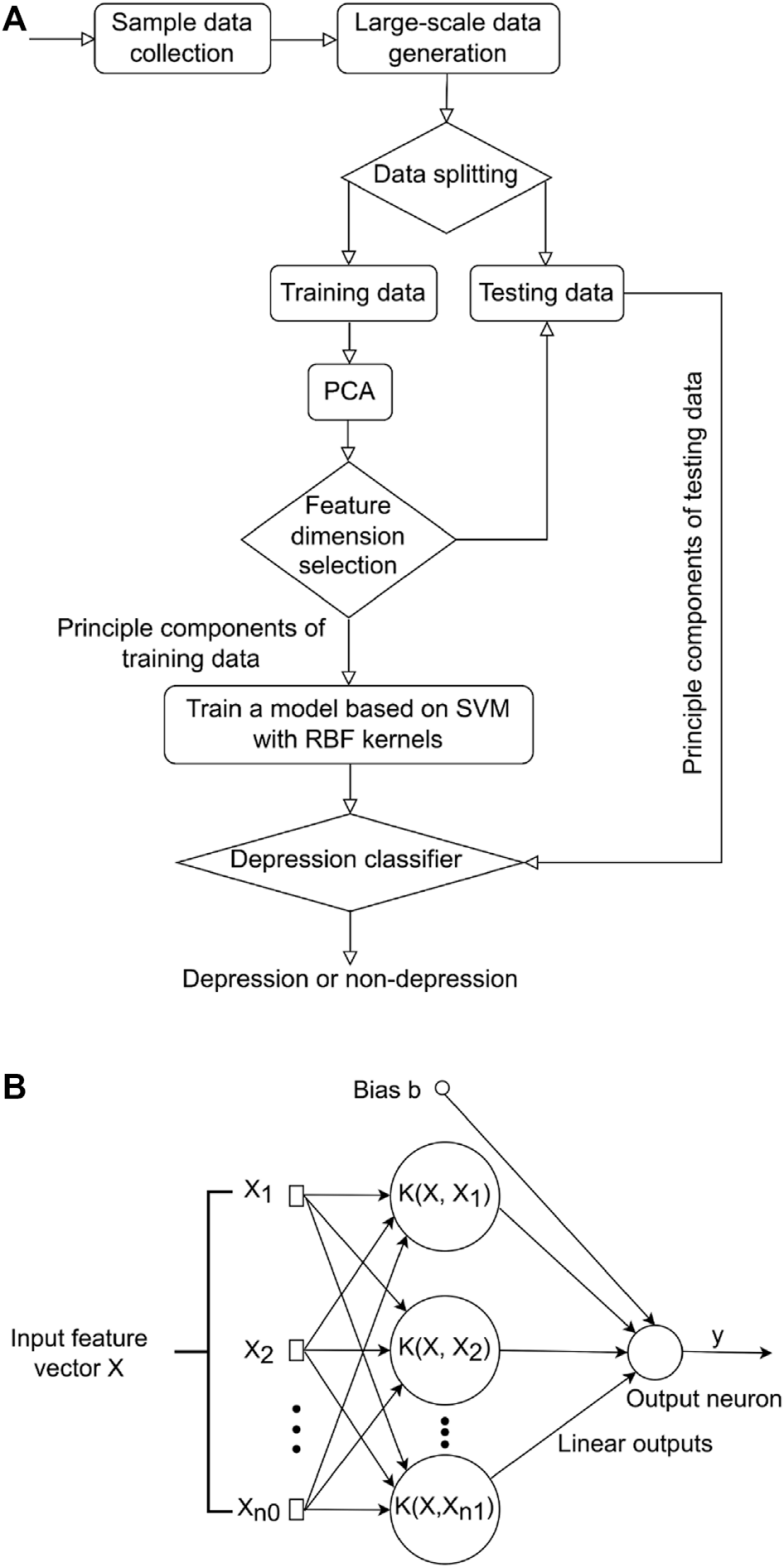
Overview of study methodology. **(A)** Flowchart of the proposed method for differentiating between those with major depression from those without in FMS patients. **(B)** Flowchart of SVM model. FMS, Fibromyalgia syndrome; SVM, support vector machine.

After a subset of gene features was selected, SVM was used to distinguish depressed and non-depressed FMS patients. PCA is a powerful tool to reduce the number of data dimensions without much loss of information (Jolliffe, 1986). A classic method taking advantages of both SVM and PCA techniques was proposed in this study (Figure 1A). More specifically, the large-scale training and testing data sets were first available through an additive model, and then split for training and testing. PCA was subsequently adopted to reduce the feature vector dimension of the training data preceding the training process. SVM was employed to learn a base model from the low dimensional feature space to recognize depression or non-depression in FMS patients.

#### 2.3.1 Generation of big-simulated gene data

The 61 samples available in the database cannot represent every possible variation of each patient. One important property of an SVM model to predict depression was the ability to generalize to the data obtained from a clinical site that was not involved in the training of the model. We adopted an additive model of embedding Gaussian noise to improve the capacity for such generalization (Formula 1). This additive model was controlled under a value of a high signal-noise-ratio (SNR) to generate a large amount of simulated data for training and testing (Wong, 2013).

$$G_{i}^{\prime }=G_{i}+N_{i}\;i=1,2,\ldots$$ (1)

#### 2.3.2 Reducing dimension of data based on PCA method

In order to reduce dimension of each sample with less loss of information, PCA technique was applied to obtain the optimal expression profiles for each sample. The sample can be expressed as a *d*-dimensional feature vector (Formula 2).

$${G=\left(g_{1}\;g_{2}\;g_{3}\;...\;g_{d}\right)}^{T}$$ (2)

where d denoted the feature vector dimension of one sample. For each sample, we had *d* observations denoting the expression profiles of multiple hub genes.

The whole processes for PCA included: i) constructing the training data set; ii) subtracting the mean from each data dimension; iii) calculating the covariance matrix; iv) calculating the eigenvectors and eigenvalues of the covariance matrix; v) determining the number of principal components; and vi) forming feature vectors for testing samples. Through PCA operation, the information redundancy of gene signals can be removed, making it easier for the model to separate depression from non-depression.

#### 2.3.3 Differentiating between those with major depression from those without in patients with fibromyalgia based on SVM model

The soft-margin SVM was constructed to differentiate depressed and non-depressed patients. Let *L* denoted the set of sample categories, and $L=\left\{1,\ldots ,l\right\}$ where *l* denoted the total number of sample categories (l = 2). This problem was considered as giving *n* labeled empirical samples.

$$\left(X_{1},y_{1}\right),\ldots ,\left(X_{n},y_{n}\right)$$

where ${\left\{X_{i}\right\}}_{i=1}^{n}$ was the PCA feature set of input samples in *R* ^*D*^ (*D* = 20), and *y*_*i*_ denoted the label of $X_{i}$ . For the classification problem; $X_{i}$ denoted the feature vector of one sample, an input PCA feature vector with the dimension of *p* (*p* = 20) and *X*_*i*_ was then transformed to a higher dimensional feature space (Chang and Lin, 2011; Nalepa and Kawulok, 2019) through a kernel function K (*X*,*Xi*) for classification as shown (Figure 1B).

Now, the goal was to find the optimal separating hyperplane that can maximize the distance between it and the nearest data points in different categories (Formula 3).

$$f\left(x\right)=w∙x+b$$ (3)

The nearest points to the hyperplane were called support vectors. To find the optimal hyperplane f (x,w), only support vectors were considered in SVM. For two linearly separated classes, the training data must satisfy the following two constraints:

$$w^{T}x_{i}+b\geq 1\;if\;y_{i}=1$$

$$w^{T}x_{i}+b\leq -1\;if\;y_{i}=-1$$ (4)

The margin of separation between two classes was thereby calculated as follows:

$$\rho =\frac{2}{\left\|w\right\|}$$

To separate depression from non-depression, the problem was to maximize the margin ρ under the constraints (Formula 4). This can be obtained by solving the optimization problem during the training process (Cortes and Vapnik, 1995). By introducing Lagrange multipliers ${\left\{\alpha_{i}\right\}}_{i=1}^{N}$ and dual transformation (Scholkopf et al., 1997; Duan and Keerthi, 2005; Chang and Lin, 2011), the category of hub genes in the test set was predicted as follows (Formula 5):

$$d_{i}=sign\left(w^{T}x_{i}\right)=sign\left(\underset{j\in SV}{\sum }\alpha_{j}d_{j}x_{j}x_{i}\right)=sign\left(\underset{j\in SV}{\sum }\alpha_{j}d_{j}k\left(x_{j},x_{i}\right)\right)$$ (5)

where *SV* denoted the set of support vectors, $k\left(x_{j},x_{i}\right)$ was a kernel function, and it was the inner product of two feature vectors. By using this kernel function, the training samples can be mapped from an input space to another feature space, which increased separation of the samples. There are two commonly used kernels for SVM in real applications, namely, linear, and radial basis function (RBF) (Apostolidis-Afentoulis and Lioufi, 2015). Generally, in the case of linearly separable data, linear kernels and RBF kernels can show similar performance, while in the case of linearly non-separable data, RBF kernels can give better prediction. Note that the dataset GSE67311 was linear non-separable data, and the number (p) of feature vector was also smaller than the number (n) of training data. For the linearly non-separable data, SVM with RBF kernels had better performance in our study. The experimental results also illustrated that the model with RBF kernels presented the better accuracy than that with linear kernels.

To obtain a more robust model with RBF kernels for the prediction of depression, K-fold cross-validation and grid search were implemented to obtain the optimal values of *gamma* and the penalty parameter (*C*). In the implementation, the precision was observed for 6 splits and 6 repeats. For each iteration, the training dataset (*trainSetAll*) was split into 6 folds, where 5 sets were served as the *trainSet*, and the remaining 1-fold was served as the *testSet*. The prediction accuracy of the model was calculated on the *testSet* in each iteration. Finally, *gamma* and *C* were obtained with the highest cross-validation accuracy. The best *gamma* and *C* were then used to train the whole training set, and the final model was generated to predict the whole testing set.

**Input**: *trainSetAll*, *type*, *kernel, m1, m2*
**Output**: *bestC* and *bestGam*
**begin**
*(trainSet, testSet) <- KfoldSplit (trainSetAll);*
*bestAccuracy = 0*;
*bestC = 0;*
bestGam = 0;
**for** *c= 2^(-m1):2^(m1)*
   **for** *s = 2^(-m2):2^(m2)*
    *model<- SVMtrain (kernel, trainSet, bestC,bestGam);*
     *acc<- SVMtest (model, testSet);*
    **if** (*acc>bestAccuracy*)
      *bestAccuracy = acc; bestC = c; bestGam = s;*
    **end**
**end**
**end**
**end**

### 2.4 Statistical analysis

The co-expression analysis and SVM model construction were conducted using MATLAB R2022a. The statistical analysis was performed by IBM SPSS Statistics 27.0 software. Chi-square test was used to determine if genes were more disrupted or invoked in FMS patients. The normality of the data was checked first. The independent sample *t*-test was applied to exam the statistical significance for Rsum values. Significant differences were found at *p*-value < 0.05.

## 3 Results

### 3.1 Structural Co-expression pattern and galaxy

**FIGURE 2:**
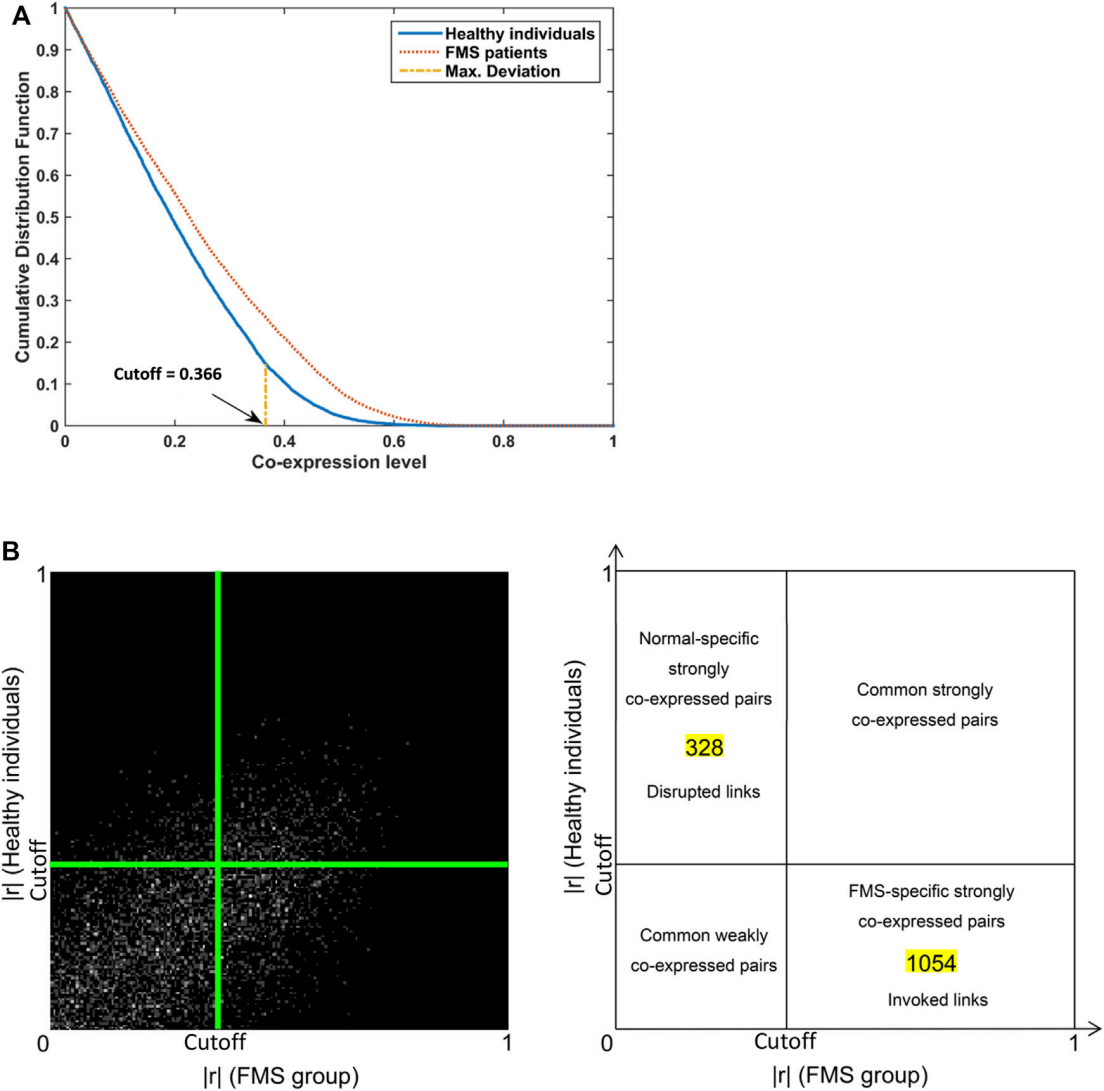
Co-expression patterns for genes involved in pain signaling pathway. **(A)** Cumulative distribution plots in the FMS and healthy individuals. The FMS-specific cutoff point 0.366 was identified. **(B)** Co-expression galaxy (left) and four regions (right) partitioned by the cutoff point. Each absolute correlation coefficient (|r|) was represented by one white dot in the galaxy. More dots demonstrated that more correlation coefficients located in that region. FMS, Fibromyalgia syndrome.

We calculated the correlation coefficients of 114 pain pathway genes in the FMS and healthy individuals, respectively. Two-sample KS test identified the significant difference in these two cumulative distributions with *p*-value < 0.001 for the maximum deviation D = 0.11 > D_*critical*_ = 0.05. The FMS-specific cutoff point, 0.366, was identified at the maximum deviation (Figure 2A). The co-expression galaxy was plotted and partitioned into four regions: i) Disrupted links; ii) Common strongly co-expressed pairs; iii) Invoked links; and iv) Common weakly co-expressed pairs (Figure 2B). From the results, we observed that links were more likely to be invoked than disrupted in FMS group, Chi-square test, *p* < 0.001.

### 3.2 Identification and annotation of hub gene features

**FIGURE 3:**
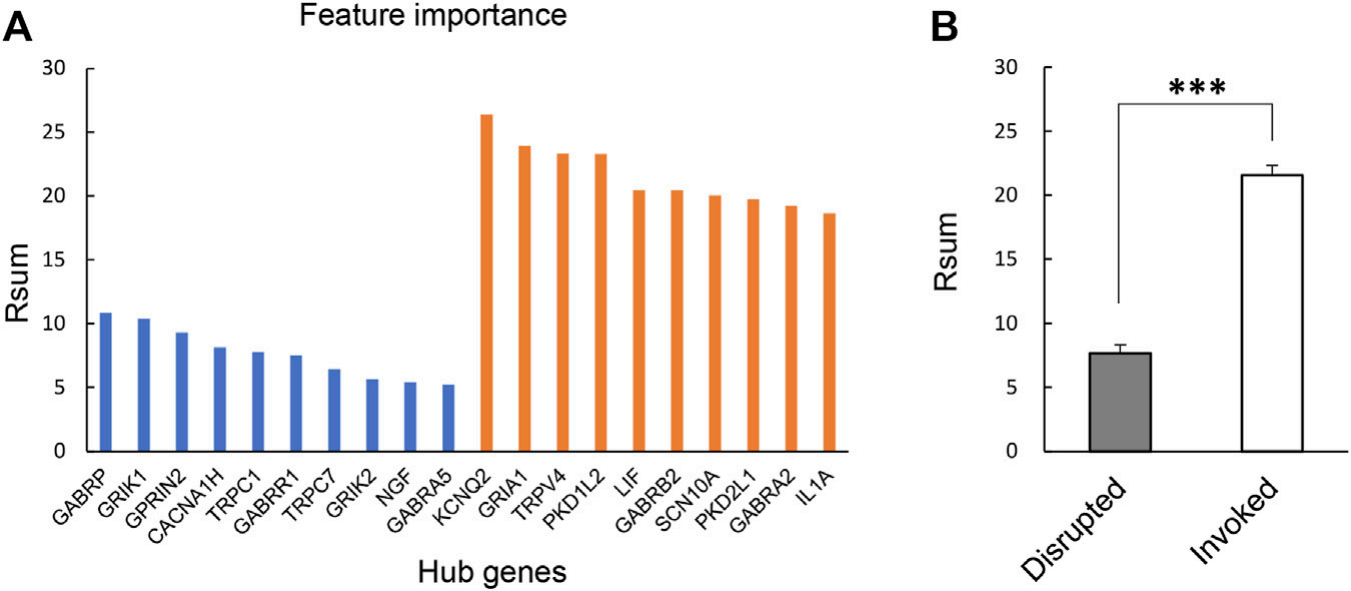
Twenty hub gene features. **(A)** Feature importance of selected 20 hub genes based on Rsum values. The blue bar referred to the hub genes from disrupted links. The orange bar referred to the hub genes from invoked links. **(B)** Rsum values for hub genes. The bar chart presents the results as the mean of 10 disrupted and 10 invoked genes. ***p < 0.001, independent sample *t*-test. Rsum, sum of |r| values.

There were 328 disrupted and 1,054 invoked links, of which involved genes were strongly co-expressed in the healthy individuals or FMS group (Figure 2B). We further explored the hub genes with the highest degree of connectivity in each group. The most important features contributing to the model were selected based on the Rsum values, including top 10 invoked and top 10 disrupted hub genes (Figure 3A). The Rsum values were significantly larger for invoked links than disrupted links (*t*-test, *p* < 0.001), indicating the important role of invoked hub genes in the development of FMS (Figure 3B).

Four major significant biological function groups were found to annotate the selected hub gene features by ClueGO in Cytoscape (Table 1; Supplementary Figure S1A): i) Ion channel complex, adjusted *p*-value < 0.001, 13 involved genes; ii) TRPs channels, adjusted *p*-value < 0.001, 3 involved genes; iii) Sodium channel activity, adjusted *p*-value < 0.001, 5 involved genes; and iv) Transmitter-gated ion channel activity involved in regulation of postsynaptic membrane potential, adjusted *p*-value < 0.001, 9 involved genes. Each major group was composed of one or several terms/pathways. The group of transmitter-gated ion channel activity involved in regulation of postsynaptic membrane potential had the most terms/pathways, and the percentage was the highest, 86.05% (Supplementary Figure S1B). The results demonstrated that these pain-related hub genes had important biological functions.

**TABLE 1:** Functional annotation for the selected 20 hub genes.

Biological function	Adjusted *p*-value	Involved genes
Ion channel complex	2.87E-25	CACNA1H, GABRA2, GABRA5, GABRB2, GABRP, GABRR1, GRIA1, GRIK2, KCNQ2, PKD2L1, SCN10A, TRPC1, TRPC7
TRPs channels	9.46E-8	TRPC1, TRPC7, TRPV4
Sodium channel activity	5.44E-10	CACNA1H, GRIK1, GRIK2, PKD2L1, SCN10A
Transmitter-gated ion channel activity involved in regulation of postsynaptic membrane potential	3.06E-12	GABRA2, GABRA5, GABRB2, GABRP, GABRR1, GRIA1, GRIK1, GRIK2, NGF

### 3.3 Differentiating between those with major depression from those without using SVM model

**FIGURE 4:**
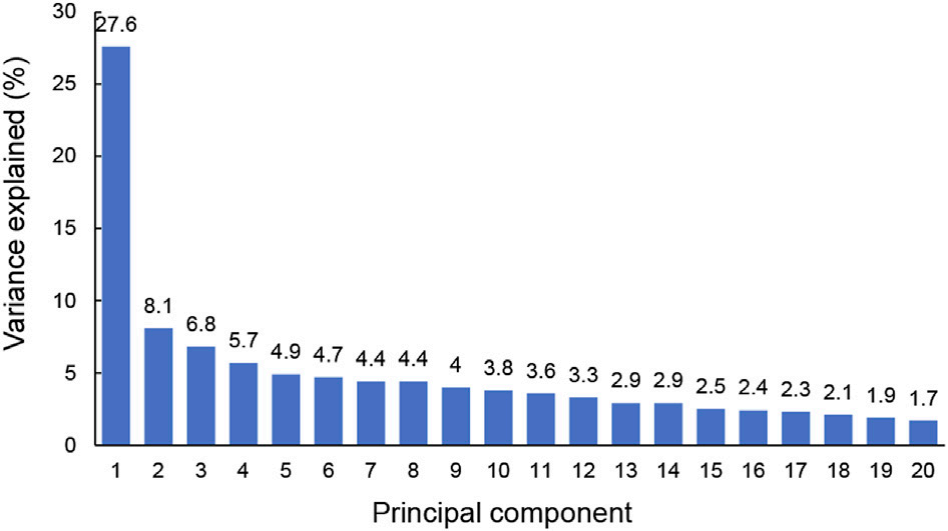
The explained variance of principal components for original training samples. The sum of the first 16 principal components for variance explained could achieve more than 90%.

**FIGURE 5:**
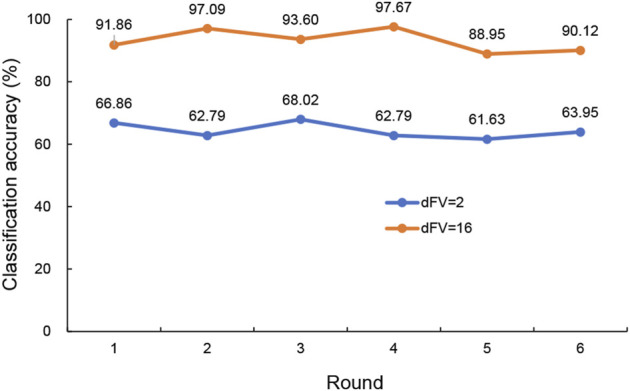
The predicted accuracy of the trained classifier based on SVM with RBF kernels for dFV values of 2 and 16. The blue curve was the accuracy for dFV = 2, and the orange curve represented the accuracy for dFV = 16. SVM, support vector machine; RBF, radial basis function; dFV, dimension of feature vector.

In this simulation, PCA was applied to 20 hub genes with expression measurements for original training samples. To get a view for the dimension of the data, the proportion of the variance within each principal component was shown (Figure 4). The results demonstrated that 16 components were needed to retain more than 90% of the original variance. In such a way, PCA reduced the dimension of the training samples from 20 to 16. The similarities and differences of the feature vectors in terms of the PCA representation could be easily highlighted due to the orthogonality properties of PCA eigenvectors. In this simulation, the trained model with the dimension of feature vector (dFV) of 16 was chosen automatically (Figure 4). We also plotted the prediction accuracy when dFV was set to 2, and the accuracy was very low due to more information lost. By contrast, the best model with dFV of 16 achieved superior performance with the average accuracy of 93.22% to differentiate between those with major depression from those without (Figure 5).

**FIGURE 6:**
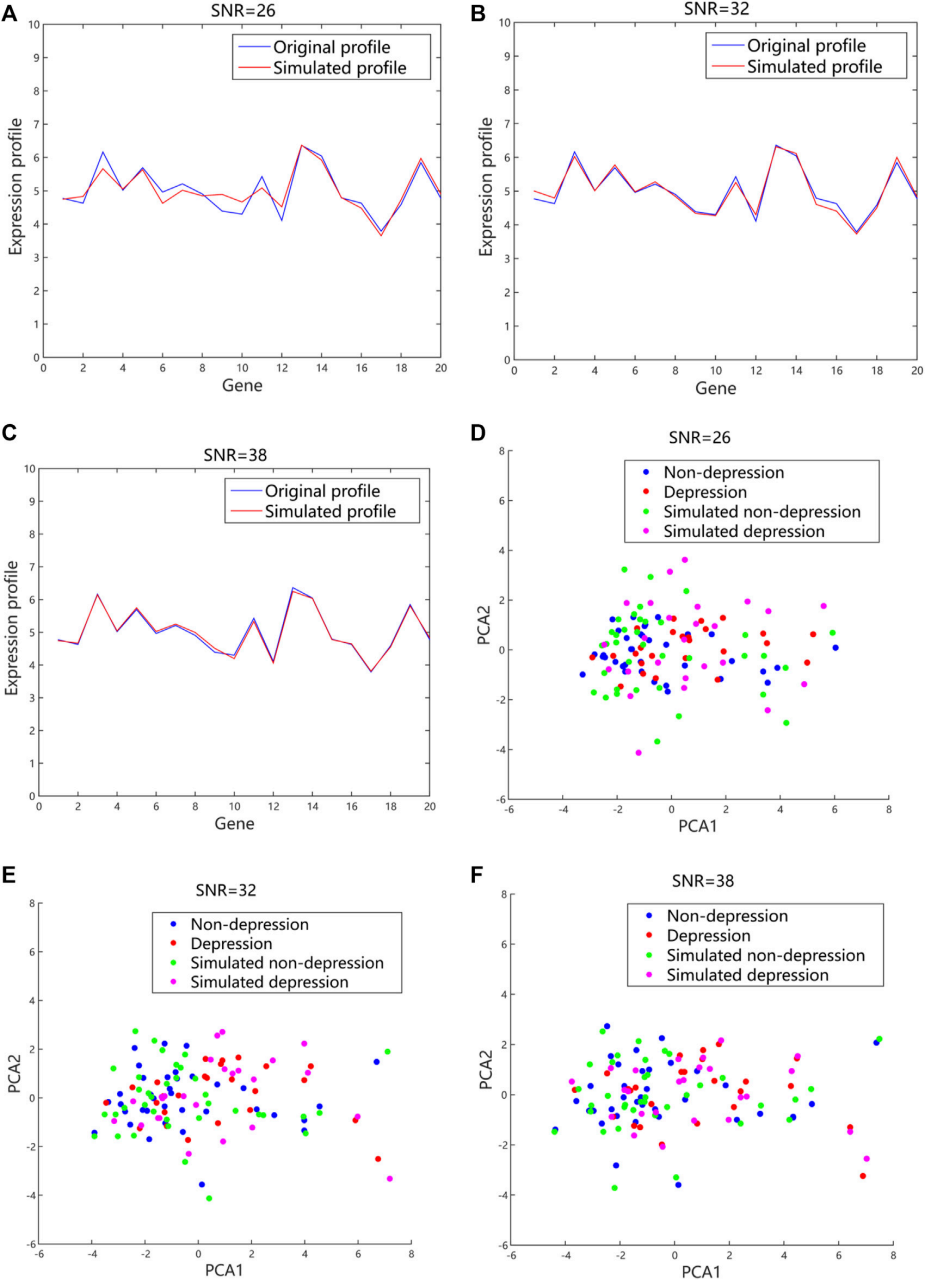
The generated gene expression data and distributions in 2D PCA space with different values of SNR based on the additive model. **(A)**, **(B)**, and **(C)** The representative simulated expression profiles with SNR values of 26, 32 and 38, respectively. The blue lines referred to the original expression profiles from microarray datasets. The red lines were the simulated expression profiles. **(D)**, **(E)**, and **(F)** The representative distributions with SNR values of 26, 32 and 38, respectively. The blue and red dots represented the original data from microarray datasets. The green and purple dots were the simulated data. 2D, 2-dimension; PCA, principal component analysis; SNR, signal-noise-ratio.

The simulated expression profiles and their distributions in 2D PCA space with different values of SNR were shown (Figure 6). The fluctuation of gene expression profile was quite large with a small value of SNR when compared to a large value. The data were dispersed randomly due to the large noises introduced. By contrast, the generated expression profiles had small fluctuation and regular distribution when a higher value of SNR was used. Usually, the larger the SNR is, the smaller the noise mixed in the signal is. However, if SNR is too large, the generated data are quite close to the original data, and difficult to simulate more possibilities. According to our simulated data, the quality of the generated data was better with SNR value of 30 dB–40 dB compared to the value of SNR between 20 dB and 30 dB. To strike the balance between the fidelity and generalizability, the SNR was set to 32 in our proposed method.

**FIGURE 7:**
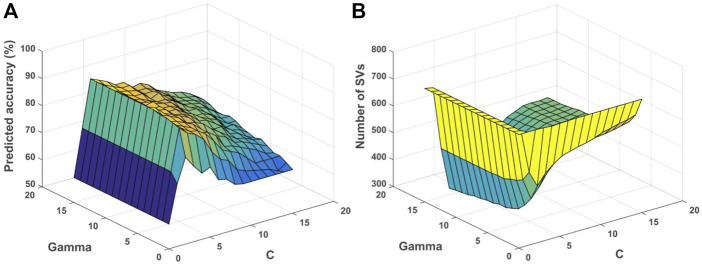
Relationship among parameters, predicted accuracy and support vectors for the depression prediction. **(A)** Relationship among *gamma*, *C* and predicted accuracy. **(B)** Relationship among *gamma*, *C* and number of SVs. SVs, support vectors.

The relationship among *gamma*, *C* and accuracy was visualized (Figure 7A). It can be observed that the value of *gamma* was larger, and the training accuracy can be higher. However, it would influence the generalization performance of each RBF kernel with the corresponding support vectors (SVs), and lead to overfitting for the training data. For unknown samples, the classification performance would be poor. By contrast, if the value of *gamma* was smaller, the smoothing effect of RBF was larger. It was difficult to obtain higher classification accuracy as shown (Figure 7A). Note that the penalty parameter *C* denoted the error intolerable of the model. The smaller the *C* was, the less fitting it was. By contrast, if *C* was too large, it was easy to make the model overfitting. Therefore, the generalization performance was poor if *C* was too large or too small as shown (Figure 7A). The relationship among *gamma*, *C* and the number of SVs was shown (Figure 7B). The smaller the *gamma* was, the number of SVs was larger, while the number of SVs would influence the time of SVM model training and testing.

**FIGURE 8:**
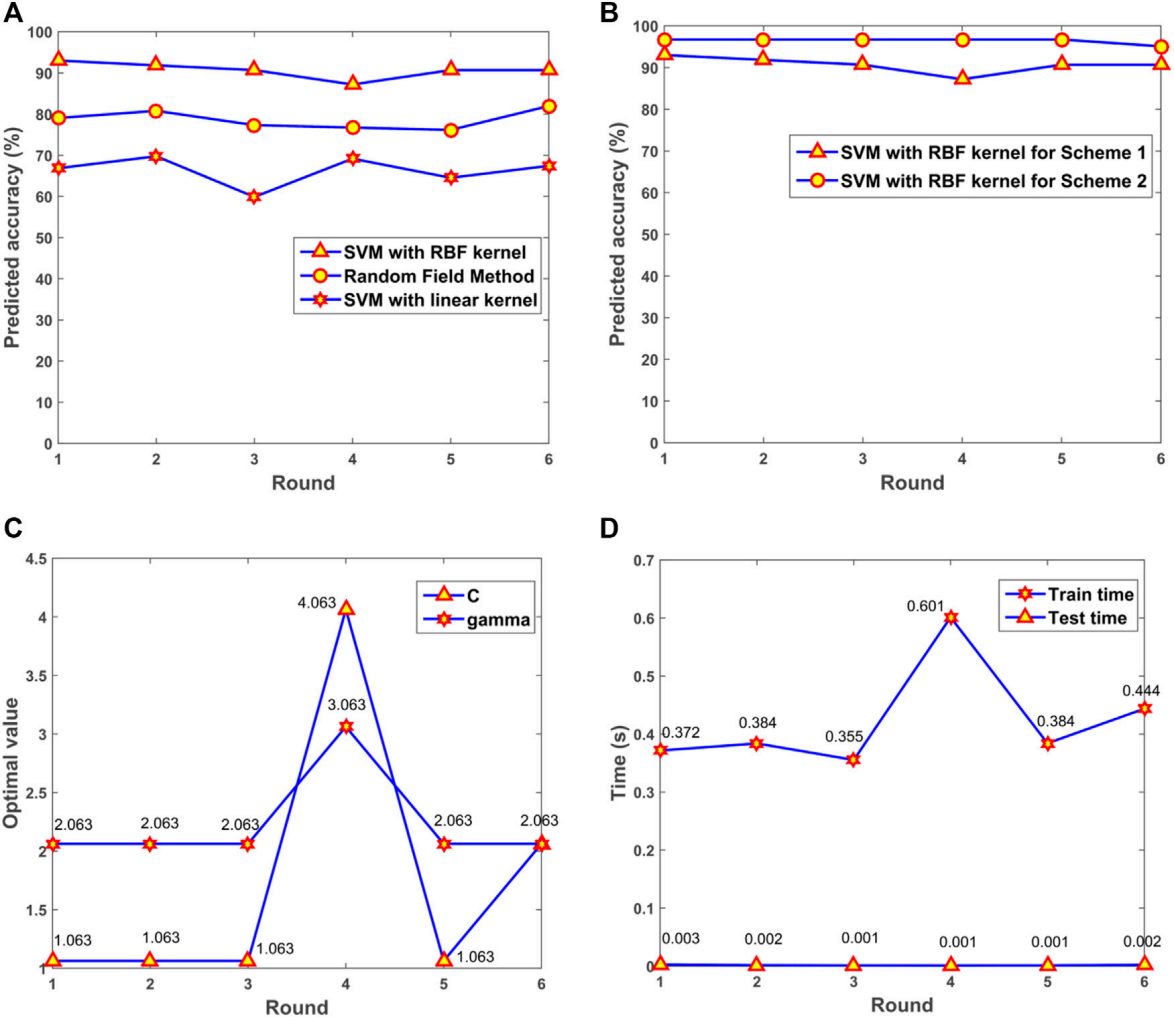
The performance of the model. **(A)** Predicted accuracy of the model with different kernels. **(B)** Predicted accuracy with different cross-validation schemes. **(C)** The optimal *gamma* and *C* in the 6-fold cross-validation. **(D)** Consuming time for training and testing processes. SVM, support vector machine; RBF, radial basis function.

The performance for SVM with RBF kernels and linear kernels was evaluated. Random Field based method was also applied for the prediction of depression in patients with Fibromyalgia (Criminisi et al., 2011; Karpathy, 2011). The results illustrated that the model with RBF kernels presented the optimal performance among the three algorithms (Figure 8A). For the SVM model with RBF kernels, two multifold cross-validation schemes were designed. In Scheme 1, all samples including original and synthesized data were split randomly for training and testing. In Scheme 2, synthesized data were used for training the SVM model, and original data were adopted for testing. The prediction accuracy was presented (Figure 8B). From the results, we can see that the trained model had a robust and consistent performance. The optimal values of *gamma* and *C* for the 6-fold cross-validation were obtained (Figure 8C). The consuming time during the training and testing processes for the 6-fold cross-validation was shown (Figure 8D). The simulation results demonstrated that the prediction was very fast, and the 172 samples only took 0.002 s for prediction.

## 4 Discussion

In this study, we investigated the feasibility of differentiating FMS patients with major depression from those without depression using microarray data by applying the support vector machine model. Of importance, the differentiation was based on gene expression levels. This is distinct from conventional clinical practice, where major depression is identified clinically without the aid of objective biological markers.

The functional annotation showed that the pain-related hub genes had important biological functions. Thirteen hub genes were involved in ion channel complex: CACNA1H, GABRA2, GABRA5, GABRB2, GABRP, GABRR1, GRIA1, GRIK2, KCNQ2, PKD2L1, SCN10A, TRPC1, and TRPC7 (Table 1). Dysfunction in the excitation/inhibition balance could lead to the major depression (Fee et al., 2017). Ion channels play a vital role in regulating the excitability, network activity and plasticity, which can alter the GABAergic and glutamatergic neuron excitability and firing, to change the excitation/inhibition balance in microcircuits (Eren-Koçak and Dalkara, 2021). Drugs targeting ion channels, including voltage-gated Na^+^ (VGSCs) and Ca^2+^ channels (VGCCs), are the major treatment for chronic neuropathic pain (Markman and Dworkin, 2006). Hub genes of TRPC1, TRPC7 and TRPV4 were found to be involved in TRPs transport extracellular Ca^2+^ to cytosol process (Table 1). Transient receptor potential (TRP) is a kind of cation channels expressed in non-excitable and excitable cells (Duitama et al., 2020). TRPs located in plasma membrane could help Na^+^, K^+^, Ca^2+^, and Mg^2+^ ions, and trace metal ions into the cells (Nilius and Owsianik, 2011). Some subfamily members that highly expressed in neurons and microglia could mediate the neuropathic pain (Haraguchi et al., 2012). As TRP channels are related to intracellular calcium regulation, signaling and painful stimuli transduction, they are regarded as promising targets to treat neurodegenerative diseases and pain (Duitama et al., 2020). TRP channels play a vital role in nociceptive, neuropathic, and inflammatory pain, owing to various family members participated in pain pathways (Hung and Tan, 2018). Another five hub genes, including CACNA1H, GRIK1, GRIK2, PKD2L1, and SCN10A, were associated with sodium channel activity (Table 1). Sodium channel is one of Voltage-gated ion channels that are very important in the electrical signaling of cells (Kandel et al., 2000; de Lera Ruiz and Kraus, 2015). Genetic and functional studies found that peripheral sensory neurons expressed sodium channels were related to human pain disorders, which can be regarded as targets for the development of new analgesics (Dib-Hajj et al., 2017). Nine genes of GABRA2, GABRA5, GABRB2, GABRP, GABRR1, GRIA1, GRIK1, GRIK2, and NGF were related to Transmitter-gated ion channel activity involved in regulation of postsynaptic membrane potential (Table 1). The transmitter-gated ion channels consist of multiple subunits of membrane-spanning receptors responsible for rapid signal transduction (Barnard, 1992). These important hub genes were selected for SVM model construction to predict depression in FMS patients.

Depression is a serious mental disorder characterized by severe and persistent low mood, negative cognitive effects and behavioral symptoms (Costi et al., 2021). FMS and depression have a bidirectional relationship, where worsening of one condition exacerbates the other, and *vice versa* (Chang et al., 2015). A number of the selected hub genes in our study have been previously reported to be related to FMS or depression, including GRIK1&2, NGF, KCNQ2, GRIA1, TRPV4, IL1A, and GABAA receptors. The hub gene was defined as the gene with the highest degree of connectivity in the co-expression network, which may play important roles in the development and characteristics of diseases, e.g., pain and depression. The expression levels of GRIK1 and GRIK2 were reported to be higher in the female patients with major depressive disorder (Gray et al., 2015). The upregulation of brain-derived neurotrophic factor mediated by NGF participates in the pathophysiological processes, leading to long-term neuroplastic changes in persistent chronic pains, e.g., fibromyalgia (Sarchielli et al., 2007). The KCNQ2/3 potassium channel is regarded as a novel treatment target for depression from preclinical studies (Costi et al., 2021). It was reported that glutamate receptors, e.g., GRIA1, may be deregulated in fibromyalgia patients (Garcia-Quintanilla and Miranzo-Navarro, 2016). TRPV4 is a kind of calcium-permeable non-selective cation channel mediating various disease states, and animals lacking TRPV4 had the decreased depression-like behavior (White et al., 2016). It was reported that significantly decreased IL1A expression was identified in depression cases compared to controls (Morrison et al., 2019). The increased GABAA receptor concentration was found in FMS compared with controls, which may lead to pain symptoms and imbalance between neuronal excitation and inhibition in FMS (Pomares et al., 2020). Our study also identified additional hub genes that have not been previously reported, such as CACNA1H and SCN10A. This discovery of hub genes that could differentiate between those with major depression from those without was based on the method that utilized both SVM and PCA techniques with a relatively high average accuracy.

The SVM studies using microarray data to select features for differentiating between those with major depression from those without are scarce. An SVM algorithm based on nuclear magnetic resonance metabolomics was developed to diagnose depression (Zheng et al., 2017). Electroencephalography recordings were applied to construct SVM classifier to predict escitalopram treatment outcome in depressed patients with the accuracy from 79.2% to 82.4% (Zhdanov et al., 2020). Moreover, diffusion-weighted neuroimaging and graph theory were used for SVM model construction to separate depressed from healthy individuals with 71.88% general accuracy (Sacchet et al., 2015). Compared to the above-mentioned methods, our current approach has the following potential advantages: i) based on the expression profiles of selected hub genes that are related to FMS or depression, making the features in SVM model more relevant to depression diagnosis in FMS patients; and ii) combination of SVM and PCA techniques to construct the model, achieving a higher accuracy of 93.22% to differentiate between those with major depression from those without.

This study has several limitations. One of these was the relatively small sample size. However, a number of other published studies using SVM for clinical medicine had even smaller sample sizes, e.g., 55 participants (Wu et al., 2018), and 32 participants (Sacchet et al., 2015). In order to address this problem, we added Gaussian noises in each gene profile of one patient by controlling SNR (Wong, 2013) to obtain some simulated data in order to represent every possible variation of each patient. A second limitation was that although we applied various measures to control the overfitting during machine learning, such as Gaussian noise and hub gene features selection techniques, our model was not perfectly unbiased. A third possible limitation was that although this study generated a large amount of simulated data for training and testing, only one data set was used, and may need a strictly independent data set to validate the model. In a study using SVM classifier to predict escitalopram treatment outcome in depressed patients, also only one dataset was applied to construct the model (Zhdanov et al., 2020). Compared to their method, our model can achieve a higher accuracy to predict depression. To polish the predictive performance of our model, future studies should validate it with an independent data set that was not used in the process of model construction. Moreover, all the depressed patients belong to major depression in the dataset used in our study, however, it was no explicitly stated on the detailed diagnostic methods in the source article. Although this was most probably corresponded to major depressive disorder in the DSM V criteria. In addition, the prediction model demonstrated in this study has yet to be proven to be generalizable to other control groups, including depressed patients without FMS or with other kinds of pain. Model evaluation should be conducted independently with some other pain conditions to exam the generalizability. This evaluation may also help to identify whether the selected hub gene features in this study were specific to depressed FMS patients or whether they can be applied to other pain conditions.

The current model provides a base which can be further polished and improved by inputting additional information from patient blood samples. A computer-based interface system using single drop blood samples can be developed to help differentiate between those with major depression from those without in FMS patients based on the expression levels of the 20 hub genes. The aim of the proposed method was to help non-psychiatrists identify FMS patients with depression that is potentially clinically significant (e.g., those with major depression). These patients should then be referred to a clinical psychiatrist for formal diagnosis (e.g., different subtypes of depressive disorders) and subsequent management for their psychiatric condition. Given that depression is a common major barrier in the management of patients with FMS, and is also often clinically unrecognized, such computer-based interface systems could become very useful in aiding clinical diagnosis and management.

In conclusion, we have demonstrated a proof-of-concept pipeline for differentiating between those with major depression from those without among FMS patients. When developed into a proper clinical application, it may contribute to the diagnosis and clinical management for patients with co-existing FMS and depression. These findings would help to develop a clinical decision-making tool for data-driven, personalized optimization of diagnosing depression in patients with FMS.

## Data Availability

Publicly available data were analyzed in this study. This dataset can be found here: Microarray dataset GSE67311, from publicly available Gene Expression Omnibus (GEO) repository database.
