# Supplementary material for: Use of pain-related gene features to predict depression by support vector machine model in patients with fibromyalgia
Source: Front Genet. 2023 Mar 29;14:1026672. doi: 10.3389/fgene.2023.1026672 (PMC10090498; doi:10.3389/fgene.2023.1026672)
Supplement: Supplementary file 2 [file Image1.pdf]

Supplementary Material

A

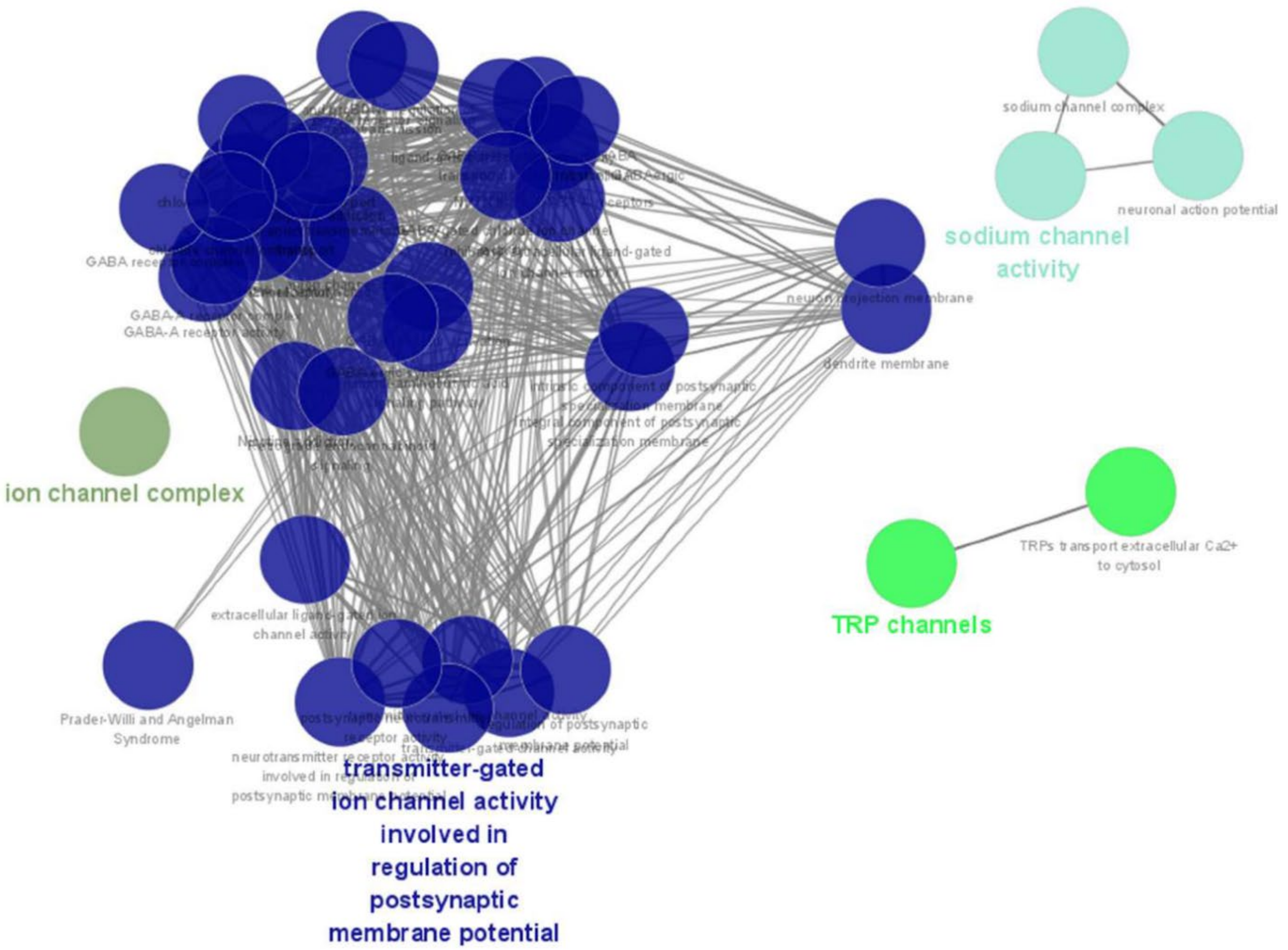
sodium channel complex
neuronal action potential
sodium channel activity
neuron projection membrane
dendrite membrane
ion channel complex
TRPs transport extracellular Ca2+ to cytosol
TRP channels
extracellular ligand-gated ion channel activity
Prader-Willi and Angelman Syndrome
transmitter-gated ion channel activity involved in regulation of postsynaptic membrane potential

B

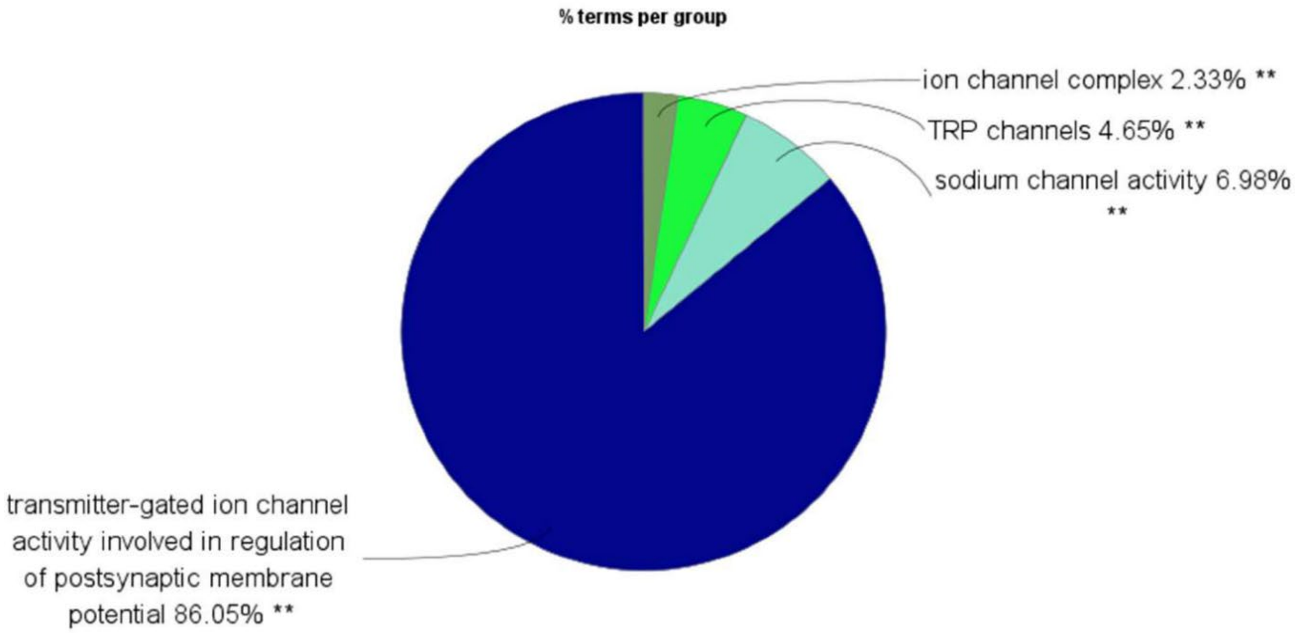
% terms per group
ion channel complex 2.33% **
TRP channels 4.65% **
sodium channel activity 6.98% **
transmitter-gated ion channel activity involved in regulation of postsynaptic membrane potential 86.05% **

Supplementary Figure 1. ClueGO annotation for the top 20 hub genes in Cytoscape. (A) Functionally grouped networks of terms/pathways. Four major groups were found with different colors. (B) Pie chart with groups based on the percentage (%) of terms/pathways per group. **Adjusted p-value<0.001.
